# The impact of loco-regional treatment modality on the outcomes in breast cancer patients younger than forty years of age

**DOI:** 10.1186/s12885-024-12325-3

**Published:** 2024-05-17

**Authors:** Rimoun R. Boutrus, Yasser A. Abdelazim, Toka Mohammed, Mohammed Bayomy, Maher H. Ibraheem, Alaadin Hussein, Medhat El Sebaie

**Affiliations:** 1 Radiation Oncology Department, National Cancer Institute, Cairo University, 1 Kasr El Aini Street, Fom El Khalig, Cairo, 11796 Egypt; 2 Department of Clinical Oncology, Zagazig University, Zagazig, Egypt; 3 Surgical Oncology Department, Breast Division, National Cancer Institute, Cairo University, Cairo, Egypt

**Keywords:** Breast cancer in young, Loco-regional treatment, Post mastectomy irradiation, Local control

## Abstract

**Purpose:**

To determine the impact of the loco-regional treatment modality, on the loco-regional recurrence (LRR) rates and overall survival (OS) in breast cancer patients younger than 40 years.

**Methods:**

Data of 623 breast cancer patients younger than 40 years of age were retrospectively reviewed. Patients were stratified according to the locoregional treatment approach into three groups: the mastectomy group (M), the mastectomy followed by radiation therapy group (MRX) and the breast conservative therapy group (BCT).

**Results:**

Median follow-up was 72 months (range, 6-180). Two hundred and nine patients were treated with BCT, 86 with MRM and 328 with MRX. The 10-year rate LRR rates according to treatment modality were: 13.4% for BCT, 15.1% for MRM and 8.5% for MRX (*p* 0.106). On univariate analysis, T stage (*p* 0.009), AJCC stage (*p* 0.047) and Her 2 status (*p* 0.001) were associated with LRR. Ten-year overall survival (OS) was 72.7% (78.5% in the BCT group, 69.8% in the MRM group and 69.8% in the MRX group, *p* 0.072).

On Univariate analysis, age < 35 (*p* 0.032), grade III (*p* 0.001), N3 stage (p 0.001), AJCC stage III (*p* 0.005), ER negative status (0.04), Her 2-status positive (0.006) and lack of chemotherapy administration (*p* 0.02) were all predictors of increased mortality.

**Conclusion:**

For patients younger than 40 years of age, similar LRR and overall survival outcomes were achieved using BCT, M or MRX. Young age at diagnosis should not be used alone in recommending one loco-regional treatment approach over the others.

## Introduction

Breast cancer is the most common female cancer with about 11% of the cases occurring in women younger than 35 years of age (http://seer.cancer.gov/statfacts/html/breast.html). In 2020, breast cancer was the leading cause of cancer related deaths in women aged 20–39 years [1].

Young age at diagnosis is usually associated with higher incidence of nodal positivity, lymphatic vascular invasion, hormone receptor negativity, Her-2 over expression and high grade tumors [2–4].

Historically, several investigators reported higher locoregional failure (LRF) rates and inferior survival in young women diagnosed with breast cancer despite intensive treatments. Fowble and colleagues found an increased rates of LRF in women younger than 35 years when compared to women 35–50 and above 50 years (24% vs. 14% vs. 12%) [5]. In a pooled analysis of 10,709 patients enrolled in to 5 NSABP studies, the 12-year incidences of IBTR for women aged 49 years or younger, 50 to 59 years, and 60 years or older were 9.6%, 5.8%, and 5.6%, respectively [6].

Recommending one treatment modality over the others was never an easy decision in such patients. Breast Conserving Therapy (BCT) is associated with better quality of life and is favored by many patients in this age group; however, concerns arise with some reports demonstrating higher LRF rates in this age group with BCT when compared with mastectomy [7].

The problem becomes more evident in the developing countries where more women are diagnosed at young age [8, 9].

The aim of the current study is to evaluate the effect of different loco-regional treatment approaches on the loco-regional recurrence rates and overall survival in breast cancer patients younger than or equal to forty years of age.

## Materials and methods

After obtaining IRB approval of National Cancer Institute, Cairo University and deeming informed consents unnecessary given the retrospective nature of the study, we retrospectively reviewed the medical records of 870 female patients, 40 years or younger, diagnosed with invasive breast cancer and treated between January 1st, 2005, and December 31st, 2013, at the National Cancer institute, Cairo University, Cairo, Egypt. Patients with DCIS, breast non-epithelial tumors, inflammatory breast cancer, metastatic disease at initial presentation or those who received neoadjuvant chemotherapy were excluded from this study. Only, 623 patients were included in the current analysis.

Patients were treated with either modified radical mastectomy (MRM), mastectomy followed by post mastectomy radiation therapy (MRX) or breast conserving therapy (BCT).

Treatment modality was chosen based on clinical stage, physician discretion and patient preferences.

Post mastectomy radiation was given using two tangential fields to the chest wall delivering 50 Gy/25 fractions /5 weeks; a direct supraclavicular field was added for patients who had positive nodes.

Patients who were treated with breast conserving surgery were treated with post-operative radiation therapy to the whole breast using two tangential fields delivering a dose of 50 Gy/25 fractions /5 weeks. A boost of 10 Gray/5 fractions was given to all patients with negative margins. Patients with margins ≤ 2 mm or with positive margins received a boost dose of 16 Gray/8 fractions.

Regional nodal irradiation was given to 69 (33%) patients in the BCT group and 210 (64%) patients in the MRX group.

Radiation therapy was administered to stage I patients after mastectomy in case of close surgical margins (< 2 mm), presence of N1mic disease or at physician’s discretion in patients with multiple adverse pathological features (triple negative histology, lymphovascular space invasion and metaplastic differentiation).

### Statistical analysis

Continuous variables were expressed as the mean ± SD and categorical variables were expressed as a number (percentage). Percent of categorical variables were compared using Pearson’s Chi-square test or Fisher’s exact test when was appropriate. Trend of change in distribution of relative frequencies between ordinal data were compared using Chi-square test for trend. Loco-regional recurrence was defined as any ipsilateral local (in-breast, chest wall or skin) or regional (supraclavicular, infraclavicular, axillary or internal mammary nodes) recurrence. Locoregional Recurrence Free Survival (LRRFS) was calculated as the time from end of treatment to date at which Locoregional Recurrence (LRR) was detected or most recent follow-up in which Locoregional recurrence was not detected (censored). Binary logistic regression analysis was done to find independent predictors for LRR, LRRFS. A *p*-value < 0.05 was considered significant. All statistics were performed using SPSS 22.0 for windows (SPSS Inc., Chicago, IL, USA) and MedCalc windows (MedCalc Software bvba 13, Ostend, Belgium).

## Results

### Patient, treatment and tumor characteristics

The median follow up for the entire cohort was 72 months (range, 6-180). Median age at diagnosis was 37 years (range, 19–40). Eighty six (14%) patients were treated with modified radical mastectomy (MRM), 328 (53%) patients were treated with mastectomy followed by radiation therapy (MRX) and 209 (33%) patients were treated with breast conserving surgery followed by radiation therapy (BCT). Patients, tumor and treatment characteristics stratified according to the loco-regional treatment modality are summarized in Table 1.

**Table 1:** Patient, tumor and treatment characteristics stratified according to the locoregional treatment approach

Characteristics	All patients (*N* = 623)		BCT (*N* = 209)		MRM (*N* = 86)		MRX (*N* = 328)		*p*-value
	No.	(%)	No.	(%)	No.	(%)	No.	(%)	
All patients	623	(100%)	209	(33.5%)	86	(13.8%)	328	(52.6%)	
*Age (years)*									
≤ 35 years	261	(41.9%)	94	(45%)	31	(36%)	136	(41.5%)	0.359
> 35 years	362	(58.1%)	115	(55%)	55	(64%)	192	(58.5%)	
*Histopathology*									
IDC	459	(73.7%)	155	(74.2%)	64	(74.4%)	240	(73.2%)	0.511
ILC	120	(19.3%)	40	(19.1%)	17	(19.8%)	63	(19.2%)	
Mixed	34	(5.5%)	8	(3.8%)	4	(4.7%)	22	(6.7%)	
others	10	(1.6%)	6	(2.9%)	1	(1.2%)	3	(0.9%)	
*Grade*									
Grade I	69	(11.1%)	25	(12%)	1	(1.2%)	43	(13.1%)	0.004
Grade II	347	(55.7%)	222	(53.1%)	58	(67.4%)	178	(54.3%)	
Grade III	191	(30.7%)	72	(34.4%)	23	(26.7%)	96	(29.3%)	
NA	16	(2.6%)	1	(0.5%)	4	(4.7%)	11	(3.4%)	
*T*									
T1	73	(11.7%)	35	(16.7%)	14	(16.3%)	24	(7.3%)	< 0.001
T2	333	(53.5%)	119	(56.9%)	47	(54.7%)	167	(50.9%)	
T3	157	(25.2%)	40	(19.1%)	14	(16.3%)	103	(31.4%)	
T4	60	(9.6%)	15	(7.2%)	11	(12.8%)	34	(10.4%)	
*N*									
N0	186	(29.9%)	73	(34.9%)	49	(57%)	64	(19.5%)	< 0.001
N1	201	(32.3%)	72	(34.4%)	27	(31.4%)	102	(31.1%)	
N2	119	(19.1%)	40	(19.1%)	5	(5.8%)	74	(22.6%)	
N3	108	(17.3%)	22	(10.5%)	2	(2.3%)	84	(25.6%)	
Nx	9	(1.4%)	2	(1%)	3	(3.5%)	4	(1.2%)	
*AJCC stage*									
Stage I	46	(7.4%)	20	(9.6%)	12	(14%)	14	(4.3%)	< 0.001
Stage II	260	(41.7%)	98	(46.9%)	51	(59.3%)	111	(33.8%)	
Stage III	317	(50.9%)	91	(43.5%)	23	(26.7%)	203	(61.9%)	
*HR status*									
Negative	344	(55.2%)	130	(62.2%)	38	(44.2%)	176	(53.7%)	0.009
Positive	249	(40%)	68	(32.5%)	40	(46.5%)	141	(43%)	
Unknown	30	4.8%)	11	(5.3%)	8	(9.3%)	11	(3.4%)	
*HER2 status*									
Negative	170	(27.3%)	57	(27.3%)	25	(29.1%)	88	(26.8%)	0.533
Positive	70	(11.2%)	22	(10.5%)	14	(16.3%)	34	(10.4%)	
Unknown	383	(61.5%)	130	(62.2%)	47	(54.7%)	206	(62.8%)	
*Biological Subtype*									
Luminal A Like	53	(20.3%)	12	(14.3%)	9	(24.3%)	32	(22.9%)	0.278
Luminal B Like	64	(24.5%)	17	(20.2%)	10	(27%)	37	(26.4%)	
Her 2 enriched	43	(16.5%)	15	(17.9%)	8	(21.6%)	20	(14.3%)	
Triple Negative	101	(38.7%)	40	(47.6%)	10	(27%)	51	(36.4%)	
*Endocrine Therapy*									
No	370	(59.4%)	138	(66%)	42	(48.8%)	190	(57.9%)	0.011
yes	223	(35.8%)	60	(28.7%)	36	(41.9%)	127	(38.7%)	
Unknown	30	(4.8%)	11	(5.3%)	8	(9.3%)	11	(3.4%)	
*Chemotherapy*									
No	45	(7.2%)	17	(8.1%)	12	(14%)	16	(4.9%)	0.013
Yes	578	(92.8%)	192	(91.9%)	74	(86%)	312	(95.1%)	

*Abbreviations*: *BCT *Breast conserving therapy, *MRM *Modified radical mastectomy, *MRX *Mastectomy followed by post mastectomy irradiation, *HR *Hormone receptor)

Patients with adverse features (T4 tumors, N3 disease, AJCC stage III, GIII and hormone receptor negative tumors) were more likely to be treated with MRX (Table 1).

Regional nodal irradiation was given to 96 (33%) patients in BCT group and 210 (64%) patients in the MRX group.

Three hundred and twelve (95%) patients in the MRX group received adjuvant chemotherapy compared to 74 (86%) and 192 (92%) in the MRM and BCT groups, respectively (*p* 0.013).

### Locoregional recurrence for the entire population

Seventy (11%) patients experienced loco-regional recurrence in the entire cohort. The 10-year rate of LRR was 15.1%, 8.8% and 13.4% in the MRM MRX and BCT groups, respectively (*p* 0.106).

On univariate analysis, T stage (*p* 0.009), AJCC stage (*p* 0.047) and Her 2 status (*p* 0.001) were shown to have statistically significant association with LRR. Other factors included in the univariate analysis are shown in Table 2.

**Table 2:** Effect of clinicopathological features on 10-year locoregional recurrence (LRR), Distant metastasis (DM) and Overall survival (OS) in all studied breast cancer patients (*N* = 623)

	All patients	LRR		*p*-value^a^	DM		*p*-value^a^	OS		*p*-value^a^
		No.	(%)		No.	(%)		No.	(%)	
Total	623	70	(11.2%)		168	(27%)		453	(72.7%)	
*Treatment modality*										
BCT	209	28	(13.4%)	0.125	43	(20.6%)	0.033	164	(78.5%)	0.072
MRM	86	13	(15.1%)		28	(32.6%)		60	(69.8%)	
MRX	328	29	(8.8%)		97	(29.6%)		229	(69.8%)	
*Age (years)*										
≤ 35 years	261	34	(13%)	0.229	83	(31.8%)	0.021	178	(68.2%)	0.032
> 35 years	362	36	(9.9%)		85	(23.5%)		275	(76%)	
*Histopathology*										
IDC	459	50	(10.9%)	0.836	126	(27.5%)	0.887	331	(72.1%)	0.863
ILC	120	14	(11.7%)		30	(25%)		90	(75%)	
Mixed	34	4	(11.8%)		10	(29.4%)		24	(70.6%)	
others	10	2	(20%)		2	(20%)		8	(80%)	
*Grade*										
Grade I	69	7	(10.1%)	0.155	14	(20.3%)	0.001	55	(79.7%)	< 0.001
Grade II	347	33	(9.5%)		77	(22.2%)		273	(78.7%)	
Grade III	191	26	(13.6%)		71	(37.2%)		116	(60.7%)	
NA	16	4	(25%)		6	(37.5%)		9	(56.2%)	
*T*										
T1	73	13	(17.8%)	0.039	20	(27.4%)	0.019	52	(71.2%)	0.060
T2	333	40	(12%)		75	(22.5%)		254	(76.3%)	
T3	157	9	(5.7%)		49	(31.2%)		111	(70.7%)	
T4	60	8	(13.3%)		24	(40%)		36	(60%)	
*N*										
N0	186	27	(14.5%)	0.463	43	(23.1%)	0.004	145	(78%)	< 0.001
N1	201	18	(9%)		44	(21.9%)		158	(78.6%)	
N2	119	11	(9.2%)		38	(31.9%)		79	(66.4%)	
N3	108	13	(12%)		37	(34.3%)		68	(63%)	
Nx	9	1	(11.1%)		6	(66.7%)		3	(33.3%)	
*AJCC stage*										
Stage I	46	10	(21.7%)	0.047	12	(26.1%)	0.005	34	(73.9%)	0.005
Stage II	260	30	(11.5%)		53	(20.4%)		206	(79.2%)	
Stage III	317	30	(9.5%)		103	(32.5%)		213	(67.2%)	
*HR status*										
Negative	344	44	(12.8%)	0.391	90	(26.2%)	0.256	249	(72.4%)	0.099
Positive	249	23	(9.2%)		66	(26.5%)		187	(75.1%)	
Unknown	30	3	(10%)		12	(40%)		17	(56.7%)	
*HER2*										
Negative	170	25	(14.7%)	0.001	57	(33.5%)	0.006	115	(67.6%)	0.006
Positive	70	15	(21.4%)		25	(35.7%)		43	(61.4%)	
Unknown	383	30	(7.8%)		86	(22.5%)		295	(77%)	
*Endocrine Therapy*										
No	370	49	(13.2%)	0.151	100	(27%)	0.226	264	(71.4%)	0.040
Yes	223	18	(8.1%)		56	(25.1%)		172	(77.1%)	
Unknown	30	3	(10%)		12	(40%)		17	(56.7%)	
*Chemotherapy*										
No	45	8	(17.8%)	0.149	16	(35.6%)	0.178	26	(57.8%)	0.020
Yes	578	62	(10.7%)		152	(26.3%)		427	(73.9%)	

Categorical variables were expressed as number (percentage)

^a^Chi-square test; *p*<0.05 is significant

On multivariate analysis, T3 disease (HR 0.334, CI 0.143–0.784, *p* 0.012) was the only factor that had independent association with lowered loco-regional recurrence free survival Table 3.

**Table 3:** Cox regression multivariate analysis of the factors associated with locoregional recurrence free survival, distant metastases, and overall mortality

Variables	HR	(95%CI)	*p*-value
**Locoregional Recurrence Free Survival**			
T3	0.334	(0.143–0.784)	0.012
Positive HER2	1.802	(0.941–3.449)	0.076
**Distant Metastasis**			
Age > 35years	0.665	(0.490–0.904)	0.009
N3	1.827	(1.168–2.858)	0.008
MRM	1.434	(1.005–2.047)	0.047
Chemotherapy administration	0.571	(0.337–0.969)	0.038
**Overall Mortality**			
Age > 35years	0.736	(0.541-1.000)	0.050
Grade III	1.236	(0.645–2.368)	0.523
N3	2.356	(1.454–3.818)	0.001
Chemotherapy administration	0.465	(0.285–0.760)	0.002

*Abbreviations:* *MRM *Modified radical mastectomy, *HR *Hazard ratio, *CI *Confidence interval

### Distant metastases for the entire population

One hundred and sixty-eight patients (27%) developed distant metastases in the entire group. The 10-year rate of distant metastasis was 32.6%, 29.6% and 20.6% for patients treated with MRM, MRX and BCT, respectively (*p* = 0.057).

On univariate analysis, loco-regional treatment strategy (*p *0.033), age group (*p* 0.021), tumor grade (*p* 0.001), T stage (*p* 0.019), N stage (*p* 0.004), AJCC stage (*p* 0.005) and Her 2 status (*p* 0.006) all had statistically significant impact on DM rate. All factors included in the univariate analysis are presented in Table 2.

On multivariate analysis, N3 disease (HR 1.827, CI 1.168–2.858, *p* 0.008) and MRM (HR 1.434, CI 1.005–2.047, *p* 0.047) were associated with increased hazard of distant metastases Table 3.

### Overall survival

Actuarial 10-year overall survival was 72.7% for the entire population. The difference in the 10-year OS among the three treatment groups was not statistically significant (69.8% for MRM, 69.8% for MRX and 78.5% for BCT, *p* 0.072).

On univariate analysis (Table 2), factors that had statistically significant correlation with OS were age group (*p* 0.032), grade (*p* 0.001), N stage (*p* 0.001), AJCC stage (*p* 0.005), ER status (0.04), Her 2 status (0.006) and chemotherapy administration (*p* 0.02).

On Multivariate analysis chemotherapy administration (HR 0.478, CI 0.293–0.780, *p* 0.003) and age above 35 years were associated with lowered mortality Table 3.

### Patients with stage I disease

Patients with stage I disease (*n* = 46) were treated with BCT (*n* = 20), MRM (*n* = 12) or MRX (*n* = 14). On univariate analysis, the loco-regional treatment modality did not have a statistically significant effect on the 10-year LRR rate, DM rate or OS. Loco-regional recurrence rate was 41.7% in the MRM group, 7.1% in the MRX group and 20% in the BCT group (*p* 0.101). The 10-year rate of distant metastases was 33.3% in the MRM group, 35.7% in the MRX group and 15% in the BCT group (*p* 0.321). The 10-year OS rate was 66.7% in the MRM, 64.3% in the MRX group and 85%% in the BCT group (*p* 0.321). Other factors included in the univariate analysis are presented in Table 4.

**Table 4:** Effect of clinicopathological features on 10-year locoregional recurrence, Distant metastasis, and Overall survival in stage I breast cancer patients (*N* = 46)

		All patients	LRR		*p*-value^a^	DM		*p*-value^a^	OS		*p*-value^a^
			No.	(%)		No.	(%)		No.	(%)	
Total		46	10	(21.7%)		12	(26.1%)		34	(73.9%)	
*treatment modality*											
BCT		20	4	(20%)	0.101	3	(15%)	0.321	17	(85%)	0.321
MRM		12	5	(41.7%)		4	(33.3%)		8	(66.7%)	
MRX		14	1	(7.1%)		5	(35.7%)		9	(64.3%)	
*Age (years)*											
≤ 35 years		17	6	(35.3%)	0.139	6	(35.3%)	0.314	11	(64.7%)	0.314
> 35 years		29	4	(13.8%)		6	(20.7%)		23	(79.3%)	
*Histopathology*											
IDC		42	9	(21.4%)	0.772	12	(28.6%)	0.462	30	(71.4%)	0.462
ILC		3	1	(33.3%)		0	(0%)		3	(100%)	
Mixed		1	0	(0%)		0	(0%)		1	(100%)	
*Grade*											
Grade I		8	1	(12.5%)	0.777	2	(25%)	0.462	6	(75%)	0.462
Grade II		22	6	(27.3%)		4	(18.2%)		18	(81.8%)	
Grade III		15	3	(20%)		6	(40%)		9	(60%)	
Unknown		1	0	(0%)		0	(0%)		1	(100%)	
*HR status*											
Negative		34	7	(20.6%)	0.316	7	(20.6%)	0.234	27	(79.4%)	0.234
Positive		8	3	(37.5%)		4	(50%)		4	(50%)	
Unknown		4	0	(0%)		1	(25%)		3	(75%)	
*HER2*											
Negative		17	4	(23.5%)	0.503	8	(47.1%)	0.016	9	(52.9%)	0.016
Positive		5	2	(40%)		2	(40%)		3	(60%)	
Unknown		24	4	(16.7%)		2	(8.3%)		22	(91.7%)	
*Endocrine Therapy*											
* No*		37	9	(24.3%)	0.531	9	(24.3%)	0.754	28	(75.7%)	0.754
* Yes*		5	1	(20%)		2	(40%)		3	(60%)	
* Unknown*		4	0	(0%)		1	(25%)		3	(75%)	
*Chemotherapy*											
No		7	0	(0%)	0.319	0	(0%)	0.165	17	(100%)	0.165
Yes		39	10	(25.6%)		12	(30.8%)		27	(69.2%)	

Categorical variables were expressed as number (percentage)

*Abbreviations:* *BCT *Breast conserving therapy, *MRM *Modified radical mastectomy, *MRX *Mastectomy followed by post mastectomy irradiation, *HR *Hormone receptor, *LRR *Locoregional recurrence, *DM *Distant metastases, *OS *Overall survival

^a^Chi-square test; *p* < 0.05 is significant

### Patients with stage II disease

Patients with stage II disease (*n* = 260) were treated with MRM (*n* = 51), MRX (*n* = 111) and BCT (*n* = 98). On univariate analysis, no statistically significant difference was found in the LRR rate when patients were stratified according to the locoregional treatment modality. The 10-year LRR rate was 9.8% in the MRM group, 11.7% in the MRX group and 12.2% in the BCT group (*p* 0.201). Other factors found to have statistically significant correlation with LRR were Her-2 status (*p* 0.035) and chemotherapy administration (*p* 0.014).

Fifty three patients (20.3%) developed distant metastases at 10 years. No statistically significant difference in the rate of distant metastases (DM) was found when patients were stratified according to the locoregional treatment modality (27.5% in the MRM group, 22.5% in the MRX group and 14.3% in the BCT group, *p* 0.127).

The 10-year OS rate was 79.2%. Factors affecting OS on univariate analysis were grade (*p* 0.001), N stage (*p* 0.030), Her 2 status (*p* 0.001) and chemotherapy administration (*p* 0.006). All factors examined in the univariate analysis are presented in Table 5.

**Table 5:** Effect of clinicopathological features on 10-year locoregional recurrence (LRR), Distant metastasis (DM) and Overall survival (OS) in stage II breast cancer patients (*N* = 260)

		All patients	LRR		*p*-value^a^	DM		*p*-value^a^	OS		*p*-value^a^
			No.	(%)		No.	(%)		No.	(%)	
Total		260	30	(11.5%)		53	(20.3%)		206	(79.2%)	
*treatment modality*											
BCT		98	12	(12.2%)	0.201	14	(14.3%)	0.127	81	(82.7%)	0.551
MRM		51	5	(9.8%)		14	(27.5%)		40	(78.4%)	
MRX		111	13	(11.7%)		25	(22.5%)		85	(76.6%)	
*Age (years)*											
≤ 35 years		104	15	(14.4%)	0.235	28	(26.9%)	0.033	74	(71.2%)	0.009
> 35 years		156	15	(9.6%)		25	(16%)		132	(84.6%)	
*Histopathology*											
IDC		182	20	(11%)	0.974	36	(19.8%)	0.803	144	(79.1%)	0.856
ILC		62	8	(12.9%)		14	(22.6%)		49	(79%)	
Mixed		9	1	(11.1%)		1	(11.1%)		8	(88.9%)	
others		7	1	(14.3%)		2	(28.6%)		5	(71.4%)	
*Grade*											
Grade I		56	6	(10.7%)	0.111	11	(19.6%)	0.245	45	(80.4%)	0.403
Grade II		143	15	(10.5%)		25	(17.5%)		116	(81.1%)	
Grade III		57	7	(12.3%)		15	(26.3%)		43	(75.4%)	
NA		4	2	(50%)		2	(50%)		2	(50%)	
*T*											
T1		15	3	(20%)	0.230	3	(20%)	0.304	11	(73.3%)	0.456
T2		204	25	(12.3%)		38	(18.6%)		165	(80.9%)	
T3		41	2	(4.9%)		12	(29.3%)		30	(73.2%)	
*N*											
N0		129	16	(12.4%)	0.665	30	(23.3%)	0.254	101	(78.3%)	0.712
N1		131	14	(10.7%)		23	(17.6%)		105	(80.2%)	
*AJCC stage*											
Stage IIA		103	17	(16.5%)	0.042	21	(20.4%)	0.999	82	(79.6%)	0.902
Stage IIB		157	13	(8.3%)		32	(20.4%)		124	(79%)	
*HR status*											
Negative		131	18	(13.7%)	0.448	27	(20.6%)	0.594	102	(77.9%)	0.537
Positive		125	12	(9.6%)		26	(20.8%)		100	(80%)	
Unknown		4	0	(0%)		0	(0%)		4	(100%)	
*HER2*											
Negative		70	11	(15.7%)	0.035	13	(18.6%)	0.880	56	(80%)	0.733
Positive		26	6	(23.1%)		5	(19.2%)		22	(84.6%)	
Unknown		164	13	(7.9%)		35	(21.3%)		128	(78%)	
*Endocrine Therapy*											
No		143	20	(14%)	0.339	30	(21%)	0.590	110	(76.9%)	0.399
Yes		113	10	(8.8%)		23	(20.4%)		92	(81.4%)	
Unknown		4	0	(0%)		0	(0%)		4	(100%)	
*Chemotherapy*											
No		14	5	(35.7%)	0.014	2	(14.3%)	0.742	9	(64.3%)	0.176
Yes		246	25	(10.2%)		51	(20.7%)		197	(80.1%)	

Categorical variables were expressed as number (percentage)

*Abbreviations:* *BCT *Breast conserving therapy, *MRM *Modified radical mastectomy, *MRX *Mastectomy followed by post mastectomy irradiation, *HR *Hormone receptor, *LRR *Locoregional recurrence, *DM *Distant metastases, *OS *Overall survival

^a^Chi-square test; *p* < 0.05 is significant

### Patient with stage III disease

In patients with stage III disease (*n* = 317), twenty three patients were treated with MRM, 203 patients with MRX and 91 patients with BCT. The locoregional treatment strategy did not affect the rate of LRR (8.7% for MRM, 10.3% for MRX and 7.7% for BCT, *p* 0.766). On univariate analysis, Her-2 status was the only factor that had a marginally significant effect on the 10-year rate of LRR (*p* 0.058).

The 10-year rate of distant metastases was 32.5%. The Locoregional treatment modality did not have a significant impact on the rate of distant metastases (43.5% in the MRM group, 33% in the MRX group and 28.6% in the BCT group, *p* 0.381). The rate of distant metastases was significantly affected by tumor grade (*p* 0.037), Her-2 status (*p* 0.002) and chemotherapy administration (*p* 0.005).

The 10-year OS rate was 76.2% for patients with stage III disease. A non-significant difference in the OS was observed based on the Locoregional treatment approach (52.2% for MRM, 66.5% for MRX and 72.5% for BCT, *p* 0.168). Factors that were found to significantly affect OS on univariate analysis were grade (*p* 0.001), N stage (*p* 0.30), Her 2 status (*p* 0.001) and chemotherapy administration (*p* 0.006). All factors included in the univariate analysis are presented in Table 6.

**Table 6:** Effect of clinicopathological features on 10-year locoregional recurrence, Distant metastasis, and Overall survival in stage III breast cancer patients (*N* = 317)

		All patients	LRR		*p*-value^a^	DM		*p*-value^a^	OS		*p*-value^a^
			No.	(%)		No.	(%)		No.	(%)	
Total		317	30	(9.5%)		103	(32.5%)		213	(67.2%)	
*Treatment modality*											
BCT		91	7	(7.7%)	0.766	26	(28.6%)	0.381	66	(72.5%)	0.168
MRM		23	2	(8.7%)		10	(43.5%)		12	(52.2%)	
MRX		203	21	(10.3%)		67	(33%)		135	(66.5%)	
*Age (years)*											
≤ 35 years		140	13	(9.3%)	0.923	49	(35%)	0.396	93	(66.4%)	0.797
> 35 years		177	17	(9.6%)		54	(30.5%)		120	(67.8%)	
*Histopathology*											
IDC		235	21	(8.9%)	0.505	78	(33.2%)	0.560	157	(66.8%)	0.613
ILC		55	5	(9.1%)		16	(29.1%)		38	(69.1%)	
Mixed		24	3	(12.5%)		9	(37.5%)		15	(62.5%)	
others		3	1	(33.3%)		0	(0%)		3	(100%)	
*Grade*											
Grade I		5	0	(0%)	0.142	1	(20%)	0.037	4	(80%)	< 0.001
Grade II		182	12	(6.6%)		48	(26.4%)		139	(76.4%)	
Grade III		119	16	(13.4%)		50	(42%)		64	(53.8%)	
NA		11	2	(18.2%)		4	(36.4%)		6	(54.5%)	
*T*											
T1		12	0	(0%)	0.204	5	(41.7%)	0.411	7	(58.3%)	0.495
T2		129	15	(11.6%)		37	(28.7%)		89	(69%)	
T3		116	7	(6%)		37	(31.9%)		81	(69.8%)	
T4		60	8	(13.3%)		24	(40%)		36	(60%)	
*N*											
N0		11	1	(9.1%)	0.732	1	(9.1%)	0.095	10	(90.9%)	0.030
N1		70	4	(5.7%)		21	(30%)		53	(75.7%)	
N2		119	11	(9.2%)		28	(31.9%)		79	(66.4%)	
N3		108	13	(12%)		37	(34.3%)		68	(63%)	
Nx		9	1	(11.1%)		6	(66.7%)		3	(33.3%)	
*AJCC stage*											
Stage IIIA		161	12	(7.5%)	0.439	49	(30.4%)	0.722	113	(70.2%)	0.464
Stage IIIB		48	5	(10.4%)		17	(35.4%)		32	(66.7%)	
Stage IIIC		108	13	(12%)		37	(34.3%)		68	(63%)	
*HR status*											
Negative		179	19	(10.6%)	0.446	56	(31.3%)	0.192	120	(67%)	0.057
Positive		116	8	(6.9%)		36	(31%)		83	(71.6%)	
Unknown		22	3	(13.6%)		11	(50%)		10	(45.5%)	
*HER2*											
Negative		83	10	(12%)	0.058	36	(43.4%)	0.002	50	(60.2%)	0.001
Positive		39	7	(17.9%)		18	(46.2%)		18	(46.2%)	
Unknown		195	13	(6.7%)		49	(25.1%)		145	(74.4%)	
*Endocrine Therapy*											
No		190	20	(10.5%)	0.437		61 (32.1%)	0.173	126	(66.3%)	0.037
Yes		105	7	(6.7%)			31(29.5%)		77	(73.3%)	
Unknown		22	3	(13.6%)			11 (50%)		10	(45.5%)	
*Chemotherapy*											
No		24	3	(12.5%)	0.485	14	(58.3%)	0.005	10	(41.7%)	0.006
Yes		293	27	(9.2%)		89	(30.4%)		203	(69.3%)	

Categorical variables were expressed as number (percentage)

*Abbreviations:* *BCT *Breast conserving therapy, *MRM *Modified radical mastectomy, *MRX *Mastectomy followed by post mastectomy irradiation, *HR *Hormone receptor, *LRR *Locoregional recurrence, *DM *Distant metastases, *OS *Overall survival

^a^Chi-square test; *p* < 0.05 is significant

## Discussion

In the present study, 623 patients younger than or equal to 40 years of age with breast cancer were retrospectively studied to detect the impact of different treatment modalities on the rate of LRR, DM and OS. Unlike previous reports, this study identified three groups of patients based on the loco-regional treatment approach; the breast conserving surgery followed by radiation group (BCT), the modified radical mastectomy group (MRM) and the mastectomy followed by post mastectomy radiation group (MRX).

In our study the incidence of 10-year LRR among all patients was 11.2%. This incidence is slightly higher than what has been published in the POSH observational cohort study which reported a LRR incidence of 4.8% among 2882 women of 18 to 40 years with breast cancer treated in the UK between 2000 and 2008 [10]. Another study from the Department of Radiation Oncology, The University of Texas M. D. Anderson cancer center, reported an LRR rate of 19.8% among 652 young patients with breast cancer treated between years 1973 and 2006 [11]. The lower rates of LRR in the current study and the POSH analysis as compared to the M.D. Anderson results might be explained by the more contemporary patient population and treatment strategies adopted in both studies.

In the current report, the rate of mastectomy was 66% vs. 34% for breast conservation. This could be explained by the higher percentage of patients with T2-3 tumors (58%) and also reflecting both patient and physician preference while treating this young age group. However, similar LRR rates were found for the three treatment groups implying no clinical advantage in choosing mastectomy over breast conservation for this group of patients. This is consistent with data published by other investigators. Plichta, et al. studied 584 women aged ≤ 40 years with breast cancer. Median age was 37 years, and median follow-up was 124 months. When stratified by lumpectomy versus mastectomy, there was no statistically significant difference in the LRF rates. Lumpectomy LRF rates were 1% at 5 years and 4% at 10 years. Mastectomy LRF rates were 3.5% at 5 years and 8.7% at 10 years. Unfortunately, there were no clear data about the percentage of patients who received post-mastectomy radiatiotherapy [12].

Kheirelseid et al. [13] prospectively analyzed data of patients with breast cancer younger than 40 years of age, treated at Galway University hospital, Department of Surgery from 1989 to 2009. They compared the LRF rates in their patients based on the surgical treatment they received. No statistically significant difference in LRF was determined comparing those who underwent mastectomy to those who had BCT.

In our study, higher incidence of LRF was detected in the patients who had hormone receptor negative tumors (10% vs. 5.6% for patients with positive hormone receptors). Among patients with negative hormone receptors, there was a statistically significant difference in the rates of LRF when stratified according to the locoregional treatment modality (17.8% for MRM vs. 7.3% for MRX and 11.6% for BCT, *p* 0.04). The clinical implication could be an essential role for radiation therapy in this subset of patients.

We were not able to demonstrate any significant association between the locoregional treatment strategy and overall survival either on univariate or multivariate analysis in the current analysis. Five year OS was 81.4% in the BCT group vs. 72.8% in the MRM group vs. 74.8% in the MRX group (*p* 0.1).

In the recently published POSH analysis, mastectomy patients had a significantly worse OS compared with BCS (HR, 0.53; 95% CI 0.45–0.62; *P* < 0.001), however, on multivariate analysis the difference was no longer significant (HR, 0.79; 95% CI 0.61–1.03; *P* 0.081) [14].

The inherent nature of this retrospective analysis carries the risk of selection bias. Furthermore, we were not able to perform further subset analysis due to the relatively small number of recurrences.

## Conclusion

For patients younger than 40 years of age, similar LRR and overall survival outcomes were achieved using BCT, M or MRX. Young age at diagnosis should not be used alone in recommending one loco-regional treatment approach over the others.

## Data Availability

Data set used by authors to generate the results is available upon reasonable request.

## Abbreviations

AJCC: American Joint Committee on Cancer
BCT: Breast Conservative Therapy
CI: Confidence Interval
DCIS: Ductal Carcinoma Insitu
DM: Distant Metastasis
ER: Estrogen Receptor
HER2: Human Epdiermal Growth Factor Receptor 2
HR: Hazard Ratio
IBTR: Ipsilateral Breast Tumor Recurrence
IRB: Institutional Review Board
LRF: Loco-regional Failure
LRR: Loco-regional Recurrence
LRRFS: Loco-regional Recurrence Free Survival
M: Mastectomy
MRM: Modified Radical Mastectomy
MRX: Mastectomy + Radiotherapy
N: Node
NSABP: National Surgical Adjuvant Breast and Bowel Project
OS: Overall survival
SD: Standard Deviation
T: Tumor
UK: United Kingdom
